# Function of the natalisin receptor in mating of the oriental fruit fly, *Bactrocera dorsalis* (Hendel) and testing of peptidomimetics

**DOI:** 10.1371/journal.pone.0193058

**Published:** 2018-02-23

**Authors:** Shun-Hua Gui, Yu-Xia Pei, Li Xu, Wei-Ping Wang, Hong-Bo Jiang, Ronald J. Nachman, Krzysztof Kaczmarek, Janusz Zabrocki, Jin-Jun Wang

**Affiliations:** 1 Key Laboratory of Entomology and Pest Control Engineering, College of Plant Protection and Academy of Agricultural Sciences, Southwest University, Chongqing, China; 2 Insect Control and Cotton Disease Research Unit, Southern Plains Agricultural Research Center, USDA, College Station, Texas, United States of America; 3 Institute of Organic Chemistry, Lodz University of Technology, Lodz, Poland; Biocenter, Universität Würzburg, GERMANY

## Abstract

Natalisins (NTLs) are conservative neuropeptides, which are only found in arthropods and are documented to regulate reproductive behaviors in insects. In our previous study, we have confirmed that NTLs regulate the reproductive process in an important agricultural pest, *Bactrocera dorsalis* (Hendel). Hence, in this study, to further confirm the *in vivo* function of NTL receptor (NTLR) and assess the potential of NTLR as an insecticide target, RNA interference targeting *NTLR* mRNA was performed. We found that mating frequencies of both males and females were reduced by RNAi-mediated knockdown of the NTLR transcript, while there was no effect on mating duration. Moreover, we functionally expressed the *B*. *dorsalis* NTLR in Chinese Hamster Ovary (CHO) cells and was co-transfected with an aequorin reporter to measure ligand activities. A total of 13 biostable multi-Aib analogs were tested for agonistic and antagonistic activities. While most of these NTL analogs did not show strong activity, one analog (NLFQV[Aib]DPFF[Aib]TRamide) had moderate antagonistic activity. Taken together, we provided evidence for the important roles of NTLR in regulating mating frequencies of both male and female in this fly and also provided *in vitro* data on mimetic analogs that serve as leading structures for the development of agonists and antagonists to disrupt the NTL signaling pathway.

## Introduction

The oriental fruit fly, *Bactrocera dorsalis* (Hendel) (Diptera: Tephritidae), originally described from Taiwan, is one of the most destructive fruit fly pests of tropical and sub-tropical areas. It attacks over 270 host plants and causes severe economic loss and trade restrictions. Moreover, under suitable conditions, a female of *B*. *dorsalis* can lay over 3,000 eggs throughout its lifetime. Therefore, after invasion, this pest can disperse rapidly with its highly reproductive potential [1,2]. Currently, the excessive and long-term use of chemical insecticides have caused serious resistance problems [3,4]. Hence, finding alternative control strategies is urgent.

Insect G protein coupled receptors (GPCRs) are important signaling molecules for cell communication, and regulate numerous vital physiological processes in insect [5,6]. Interfering with normal GPCRs function by blocking or over stimulating their endogenous activities may disrupt normal fitness to control the pest. Hence, GPCRs are considered as potential targets for developing novel pesticides [5,7]. Natalisins (NTLs) are conserved neuropeptides, which are only found in arthropods and were documented to regulate reproductive behaviors via activating their receptors (NTLRs) in insect [8]. In our previous study, we have identified NTL in the oriental fruit fly, *Bactrocera dorsalis* (Hendel). Using a calcium reporter assay, we also identified its receptor NTLR, which is a typical GPCR [9]. Moreover, RNA interference (RNAi) mediated by double-stranded RNA (dsRNA) injection in adults confirmed that NTL regulates reproductive processes also in *B*. *dorsalis* [9]. Hence, NTLR may be a potential target for developing novel insect control agents via synthesizing NTL peptidomimetic analogs. Interestingly, the typical C-terminal FxxxRamide motif of NTLs is similar to the C-terminal FxGxRamide motif of tachykinin-related peptides (TRPs) [8,10]. TRPs are multifunctional neuropeptides, and biostable multi-Aib (α-aminoisobutyric acid) analogs of TRPs have potent oral aphicidal activity against the pea aphid *Acyrthosiphon pisum* via synthesizing [11]. The successful case of TRPs sheds lights on the development of NTL peptidomimetic analogs for this fruit fly pest.

In the current study, we investigated the effect of RNAi-mediated knockdown of NTLR on the mating behavior in *B*. *dorsalis*. Furthermore, we examined the ligand activities of *B*. *dorsalis* NTLR in a heterologous reporter system with NTL peptides and its biostable multi-Aib peptidomimetic analogs.

## Materials and methods

### Ethics statement

No specific permits were required for the insects collected in this study. No specific permissions were required for these locations/activities which the insect specimens were collected. We confirm that these locations are not privately-owned or protected in any way and the species collections did not involve endangered or protected species.

### Flies and chemicals

The stock colony of oriental fruit fly was obtained as previously described [9]. The flies were kept at 27 ± 1°C and 70 ± 5% relative humidity, and a photoperiod regime of 14 h light/10 h darkness. The NTL peptides of *B*. *dorsalis* were synthesized by Genescript (Genescript, Nanjing, China). All biostable multi-Aib peptidomimetic analogs were synthesized in Nachman’s laboratory at Southern Plains Agricultural Research Center, USDA, USA. Plasmids for transfection were prepared using the Plasmid *Plus* Midi kit (Qiagen, Valencia, CA). Cell culture reagents including DMEM/F12 medium, fetal bovine serum, fungizone and penicillin/streptomycin, and coelenterazine for aequorin functional assays were purchased from Gibco cell culture at Life Technologies (Life Technologies, Grand Island, NY). The transfection reagent (TransIt) was purchased from Mirus Bio (Mirus Bio, Madison, WI).

### Sequence analysis

Multiple sequence alignments were made by CLUSTAL X2 software [12] and formatted in JalView 2.9 [13]. Transmembrane helices were predicted using a TMHMM server (http://www.cbs.dtu.dk/services/TMHMM).

### RNA interference

T7 promoter sites were incorporated on either side of primers (forward, 5’-taatacgactcactatagggCGGTATTTACCCTTGTGGCTA-3’ and reverse, 5’-taatacgactcactatagggTGTTGTACGTATTTGGCGGAT-3’) to amplify the double-stranded RNA (dsRNA) region of NTLR precursor of *B*. *dorsalis*. The amplified dsRNA region of NTLR included 520 bp bases and was presented in S1 Fig. The amplicon was purified and verified by DNA sequencing. Then, the dsRNA was generated using a Transcript Aid T7 High Yield Transcription Kit (Thermo Scientific, Lithuania, EU) and its length was checked on a 1% agarose gel.

The 1.2 μg dsRNA-NTLR or dsRNA-GFP was mixed with lipofectamine (Invitrogen) at a volume ratio of 1:1, respectively. The mixture was injected into the body cavity of the 3-, 5- and 7- day old-adults directly between the second and third abdominal segments using a Nanoject II Auto-Nanoliter Injector (Drummond Scientific, Broomall, PA).

### RNA knockdown efficiency assay

Twenty-four and 48 h after the dsRNA injection, samples were prepared to check the RNA knockdown efficiency of NTLR. Each treatment included three independent groups of four individuals (two females and two males). Total RNA was extracted with TRIzol reagent (Invitrogen, Carlsbad, CA) and treated with RQ1 DNase I (Promega, Madison, WI) to eliminate genomic DNA, followed by phenol-chloroform extraction. First-strand cDNA was synthesized by GoScript Reverse Transcription System (Promega) for RT-PCR with random hexamers in a total volume of 20 μl according to the manufacturer's instructions.

Quantitative real-time PCR (qRT-PCR) was performed to calculate the RNA knockdown efficiency. The detailed method of qRT-PCR and primer sequences of *B*. *dorsalis* NTLR were described in our previous study [9]. Moreover, due to *B*. *dorsalis* NTLR had some conserved structures with *B*. *dorsalis* TRP receptor (TRPR), to exclude potential off target effects for TRPR, the transcript level of TRPR was also checked after injecting NTLR-dsRNA. The primer sequences of *B*. *dorsalis* TRPR were described in the previous study [14]. For data analysis, the relative expressions were determined using the 2^-ΔΔCt^ method [15]. The qRT-PCR data was analyzed by Student’s *t*-test.

### Behavioral assay

Behavioral assay was performed as previously reported [9]. Under standard laboratory conditions, virgin females and naive males were collected separately within 24 h after eclosion. Subsequently, insects were injected for three times with dsRNA as mentioned above. At 24 h after the third injections of dsRNA, the treated females were individually placed in a 4.5 cm diameter and 3.5 cm deep transparent chamber together with the control males individually. Vice versa, the injected males were individually crossed with the control females. To ensure that there was adequate time for acclimation, each pair of flies was transferred to the mating chamber at the beginning of the light photoperiod. Mating behavior was observed every 30 min over a 10 h dark photoperiod and the mating duration was recorded. Data of mating frequency was analyzed using Fisher′s exact test, and the mating duration data was analyzed using Student’s *t* test.

### Heterologous expression and functional assay

The recombinant plasmid NTLR-pcDNA3.1(+) was obtained in our previous study [9]. High-quality plasmid DNA was prepared using the plasmid MIDIprep kit (Qiagen) and was used for transient transfection. An *in vitro* calcium mobilization assay was performed using Chinese hamster ovary (CHO-WTA11) cells supplemented with aequorin and Gα16. The cells were collected 30 h later and pre-incubated with coelenterazine (Invitrogen) for the calcium mobilization assay according to the published protocols [16–18]. The luminescence-based calcium mobilization assays were measured using a TriStar^2^ LB 942 Multimode Reader (Berthold Technologies, Bad Wildbad, Germany). For pharmacological assays of the peptidomimetics, we measured the relative activities of the peptidomimetics on NTLR that were normalized by the activity of the endogenous ligand BdNTL2 (HNLPDLDALLNRYETFVPNRamide), which showed the highest activity for NTLR in our previous work [9]. We treated the CHO cells with 13 peptidomimetics (final concentration of 1μM) and measured the luminescence, then kept a 30 min incubation of the cells with the test peptidomimetics. Subsequently, these CHO cells were incubated with BdNTL2 (final concentration of 1 μM) and we measured the luminescence again. Methods for the assay and data analyses were previously described [17,18]. Briefly, the luminescence responses of the cells obtained in the first treatment with test-peptidomimetics relative to the response of BdNTL2 were regarded as agonistic activity (AG). The responses of cells in the second treatment with the BdNTL2 were regarded as the remaining activity. The sum of agonistic activity and remaining luminescence activity was subtracted from 100% to obtain the antagonistic activity (ANT). Therefore, the value for antagonistic activity discriminated antagonistic activity from the desensitization activity of agonists. All mimetics were tested at 1 μM, which is the lowest concentration that induced the maximum response in the endogenous ligand BdNTL2 for three biological replications.

## Results and discussion

### *B*. *dorsalis* NTLR

**Fig 1:**
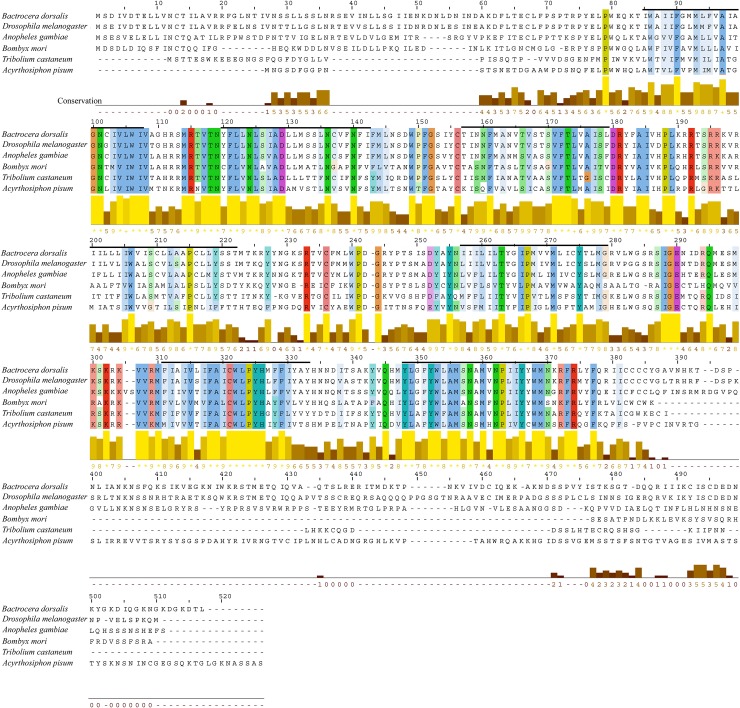
Amino acid sequence alignment of *B*. *dorsalis* NTLR with several NTLRs from other species. Bold black horizontal bars indicate transmembrane domains.

In our previous work, we have reported the sequence of *B*. *dorsalis* NTLR (accession number KU645848) [9]. Multiple sequence alignment (Fig 1) with the NTLRs of *Drosophila melanogaster* (AAA28722) [19], *Anopheles gambiae* (XP_312088) [4], *Bombyx mori* (NP_001127749) [20], *Tribolium castaneum* (EEZ99366) [21] and *Acyrthosiphon pisum* (XP_001946954) showed that NTLRs are highly conserved in insect species. Among these GPCRs, maximum similarity concerns the regions encompassing the transmembrane domains. However, the C-terminal intracellular tail diverges among different species. Overall, our analysis indicates that NTLRs are evolutionarily conserved.

### dsRNA-mediated gene-silencing of *B*. *dorsalis* NTLR

**Fig 2:**
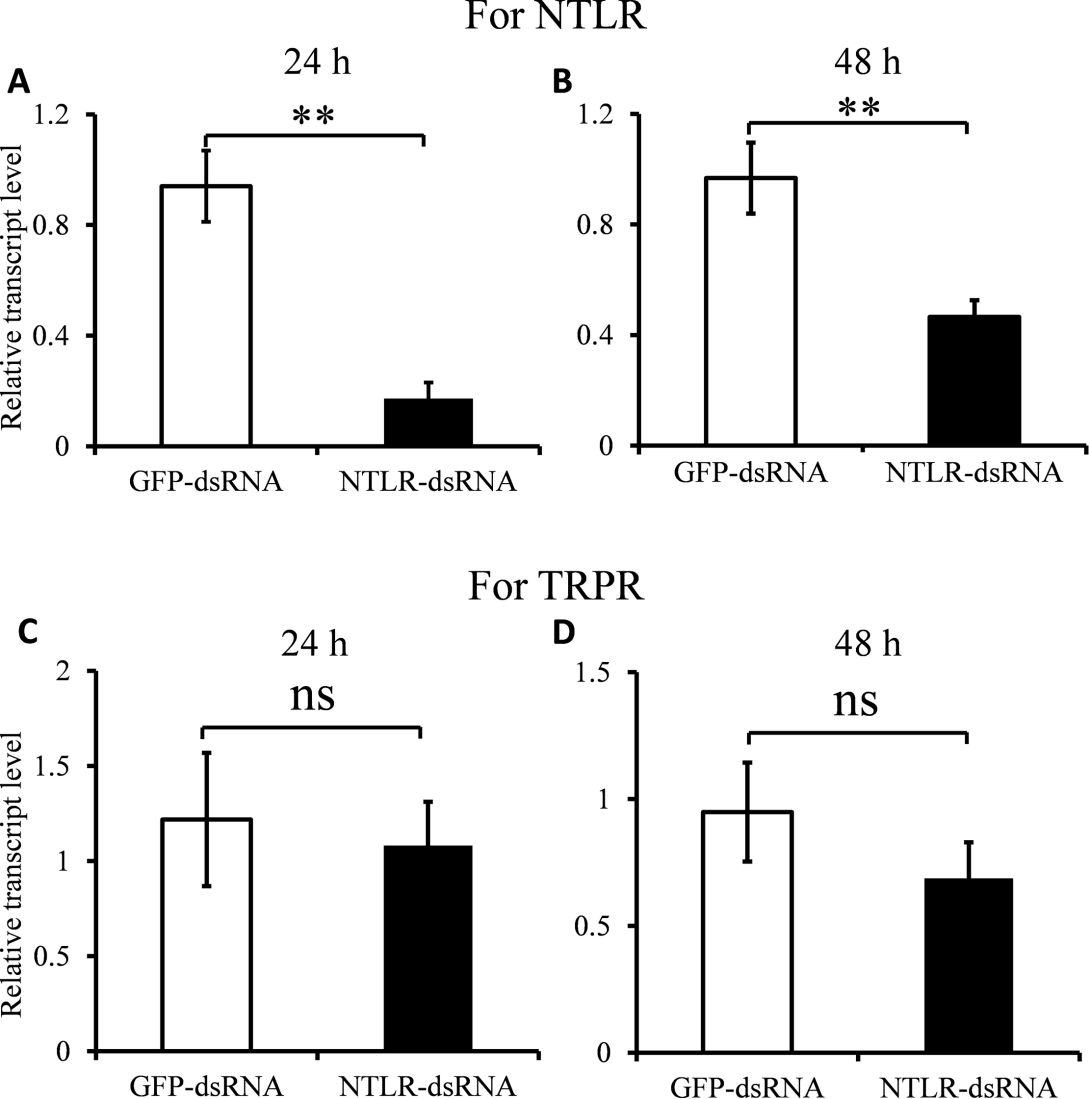
Effect of NTLR-dsRNA injection on the *B*. *dorsalis* NTLR and TRPR transcript levels. Knockdown efficiency of NTLR-RNAi was measured at 24 h (A) and 48 h (B) after the third injection of dsRNA. Similarly, effect of RNAi-mediated knockdown of NTLR on *B*. *dorsalis* TRPR transcript levels was measured at 24 h (C) and 48 h (D) after the third injection of dsRNA. Data are means ± SD, n = 3, Asterisks indicate significant differences in relative expression (**P < 0.01), Abbreviation: ns, not significant (*P* > 0.05).

**Fig 3:**
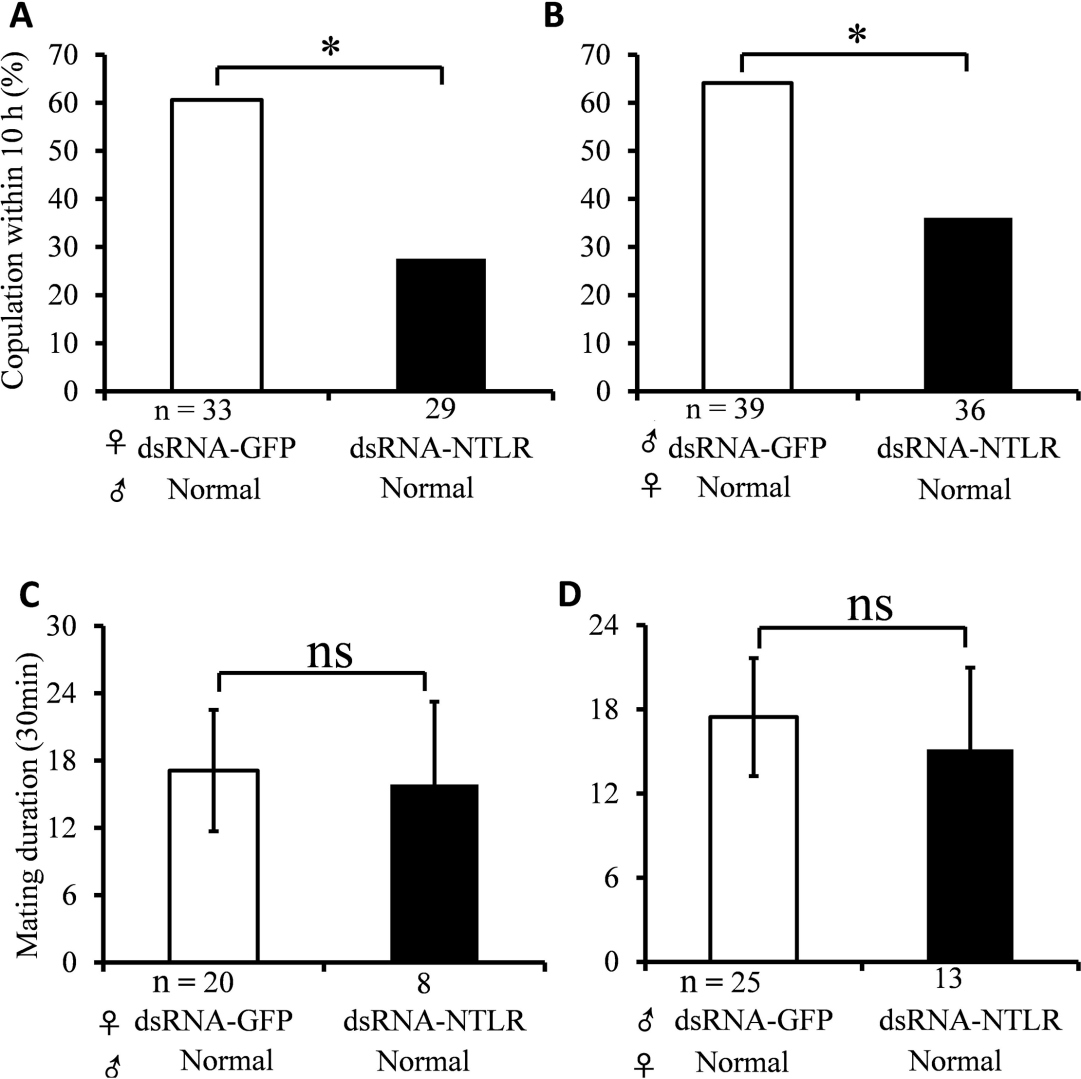
Effect of NTLR-RNAi on mating frequencies in *B*. *dorsalis*. (A) Mating frequencies of NTLR-RNAi females in a single-pair assay with untreated naive males. (B) Mating frequencies of NTLR-RNAi males in a single-pair assay with untreated virgin females. (C) Mating duration NTLR-RNAi females in a single-pair assay with untreated naive males. (D) Mating duration of NTLR-RNAi males in a single-pair assay with untreated virgin females. Asterisks indicate significant differences in relative expression (*P < 0.05). n, sample sizes. Abbreviation: ns, not significant (*P* > 0.05).

We recently reported that NTL play an important role in regulating both male and female mating frequencies in *B*. *dorsalis* [9]. In this study, to further assess the *in vivo* function of NTL signaling system in *B*. *dorsalis*, similar mating assays were performed by RNAi-mediated knockdown of the NTLR. The knockdown efficiencies were significant with 81.6% (*P* < 0.01) and 51.9% (*P* < 0.01) at 24 and 48 h post-injection of dsRNA-NTLR, respectively (Fig 2A and 2B). Moreover, to check the potential off-target effect for TRPR, the transcript levels of TRPR were measured after injecting NTLR-dsRNA. The results showed NTLR-dsRNA had no influence upon the transcript level of TRPR (*P* > 0.05; Fig 2C and 2D), though *B*. *dorsalis* NTLR has some conserved structures with *B*. *dorsalis* TRPR. As indicated by the results in mating assays, there was a significant reduction in the mating frequency of treated females after dsRNA-mediated silencing of *B*. *dorsalis NTLR* (27.6%) compared to the control (60.6%) (*P* < 0.05; Fig 3A). Likewise, the mating frequency of NTLR-dsRNA treated males (36.1%) was significantly less than flies treated with GFP-dsRNA (64.1%) (*P* < 0.05; Fig 3B). Linking our previous works that NTL regulate mating of *B*. *dorsalis* [9] and current data, we clearly prove that NTLR can be a potential target for a novel control strategy of the pest fly. Nevertheless, we found that there was no significant effect of mating duration between normally mated NTLR-dsRNA flies and control files (*P* > 0.05; Fig 3). The data showed that the mating duration of all flies could keep 7–9 h, this is in accordance with statistical data of normal flies obtained from previous study [22]. This suggests that NTLR may influence mating frequency via regulating mating desire [8].

### Activity of peptidomimetics containing multiple, sterically hindered Aib residues on *B*. *dorsalis* NTLR

Mating is a vital instinctive behavior to ensure breeding populations of insects. Blocking insect mating could lead to reduction of pest populations and in turn control the pest. As mentioned above, we found that the NTLR regulated mating frequency of *B*. *dorsalis*. This important physiological function of NTLR provides a basis for developing NTL peptidomimetic analogs to target NTLR. Hence, we *in vitro* tested the agonistic and antagonistic activities of NTL peptidomimetic analogs for *B*. *dorsalis* NTLR in a heterologous reporter system. These peptidomimetic analogs were designed via incorporating of sterically hindered α-aminoisobutyric acid. Generally proteolytic degradation restricts the use of peptidomimetic analogs capable of modulating aspects of insect physiology. Yet, the sterically hindered α-aminoisobutyric acid residue resists peptidase action [23]. This approach was considered as an idea to develop biostable peptidomimetic analogs; its potency has been demonstrated *in vivo* [11,24–27]. In our present study, thirteen peptidomimetics did not show strong agonistic and antagonistic activities (Table 1). Among these peptidomimetics, the peptidomimetic 2104[Φ1]wp-2 (TE[Aib]N[Aib]FW[Aib]NRa) had the highest agonistic activity, while it is only 10.03%. For the assay of antagonistic activity, only the peptidomimetic 2120[Φ1]wp-1 (NLFQV[Aib]DPFF[Aib]TRamide) showed antagonistic activity of 31.25%. Moreover, due to the similarity between NTL and TRP motif, we tested two TRP peptidomimetics 1888[Φ1]wp-4 and 1887[Φ1]wp-3, which had significantly aphicidal activity in the pea aphid [11]. However, the two peptidomimetics have neither agonistic nor antagonistic activities on NTLR of *B*. *dorsalis*. This may be explained by the pharmacological differences between the two receptors NTLR and TRPR, although NTL and TRP *in vitro* assay system have cross interactions [9,14].

**Table 1:** Agonistic and antagonistic activities of the peptidomimetics against BdNTLR.

Chemical ID	Sequence information	AG (%)	ANT (%)
1887[Φ1]wp-3	pEA[Aib]S[Aib]FL[Aib]VRa	-2.09 (± 1.16)	9.03 (± 14.41)
1888[Φ1]wp-4	pEA[Aib]SGFL[Aib]VRa	1.01 (± 1.70)	3.89 (± 13.74)
2098[Φ3]wp-1	TE[Aib]NPFW[Aib]NRa	5.63 (± 2.17)	-2.08 (± 7.41)
2065-SP2[Φ1]wp-1	pQE[Aib]GPFW[Aib]NRa	3.03 (± 1.43)	0.24 (± 3.31)
2131[Φ1]wp-4	KEN[Aib]PNFW[Aib]SRa	2.45 (± 0.20)	2.87 (± 2.58)
2102[Φ2]wp-2	NLFQV[Aib]D[Aib]FF[Aib]TRa	-0.16 (± 1.62)	10.95 (± 11.21)
2104[Φ1]wp-2	TE[Aib]N[Aib]FW[Aib]NRa	10.03 (± 2.14)	-21.78 (± 28.18)
2120[Φ1]wp-1	NLFQV[Aib]DPFF[Aib]TRa	-0.64 (± 0.88)	31.25 (± 2.13)
2108[Φ1]wp-1	SVR[Aib]DPTY[Aib]ARa	7.98 (± 7.60)	-12.86 (± 1.87)
2111[Φ1]wp-1	AEGD[Aib]DYFW[Aib]TRa	4.76 (± 0.73)	-4.42 (± 9.08)
2130[Φ3]wp-2	SVR[Aib]D[Aib]TY[Aib]ARa	1.31 (± 0.66)	-2.20 (± 10.29)
2069[Φ2]wp-2	pQY[Aib]DLFY[Aib]HRa	0.50 (± 0.61)	8.32 (± 6.40)
2070[Φ4]wp-4	pQE[Aib]G[Aib]FW[Aib]NRa	9.50 (± 6.82)	18.28 (± 4.37)

Data are means ± SE. AG, Agonistic activity. ANT, Antagonistic activity. Aib, α-aminoisobutyric acid.

## Conclusions

We provided evidence for an important role of NTLR in the regulation of mating process in *B*. *dorsalis* using dsRNA-mediated gene-silencing of NTLR. Thus, our results are in agreement with and complement our previous study to support the conclusion of NTL signaling plays a role in reproductive physiology. This result verified the potential of NTLR as a target for novel insecticide development against *B*. *dorsalis*. Although we were unable to identify a strong agonist and antagonist for *B*. *dorsalis* NTLR in our tests of a limited set of peptidomimetics, the screening with a heterologous cell expression system of *B*. *dorsalis* NTLR may be a useful approach to identify *B*. *dorsalis* NTLR agonists and antagonists. To find *B*. *dorsalis* NTLR specific agonists and antagonists, further expansion of the Aib-containing peptidomimetics based on NTL sequences is needed. Taken together, we believe these results can help in developing novel pest control strategies against this fruit fly, even the Tephritidae pests in the future.

## Supporting information

S1 Fig Sequence of the *B. dorsalis* NTLR cDNA and the corresponding amino acid sequence. The annealing sites of the qRT-PCR primers are denoted in bold and underlined, while the annealing sites of the primers for the RNAi-constructs are denoted in bold and dashed underlined in the cDNA sequence.
(JPG)
