# Supplementary figures and images for: Function of the natalisin receptor in mating of the oriental fruit fly, Bactrocera dorsalis (Hendel) and testing of peptidomimetics
Source: PLoS One. 2018 Feb 23;13(2):e0193058. doi: 10.1371/journal.pone.0193058 (PMC5825034; doi:10.1371/journal.pone.0193058)

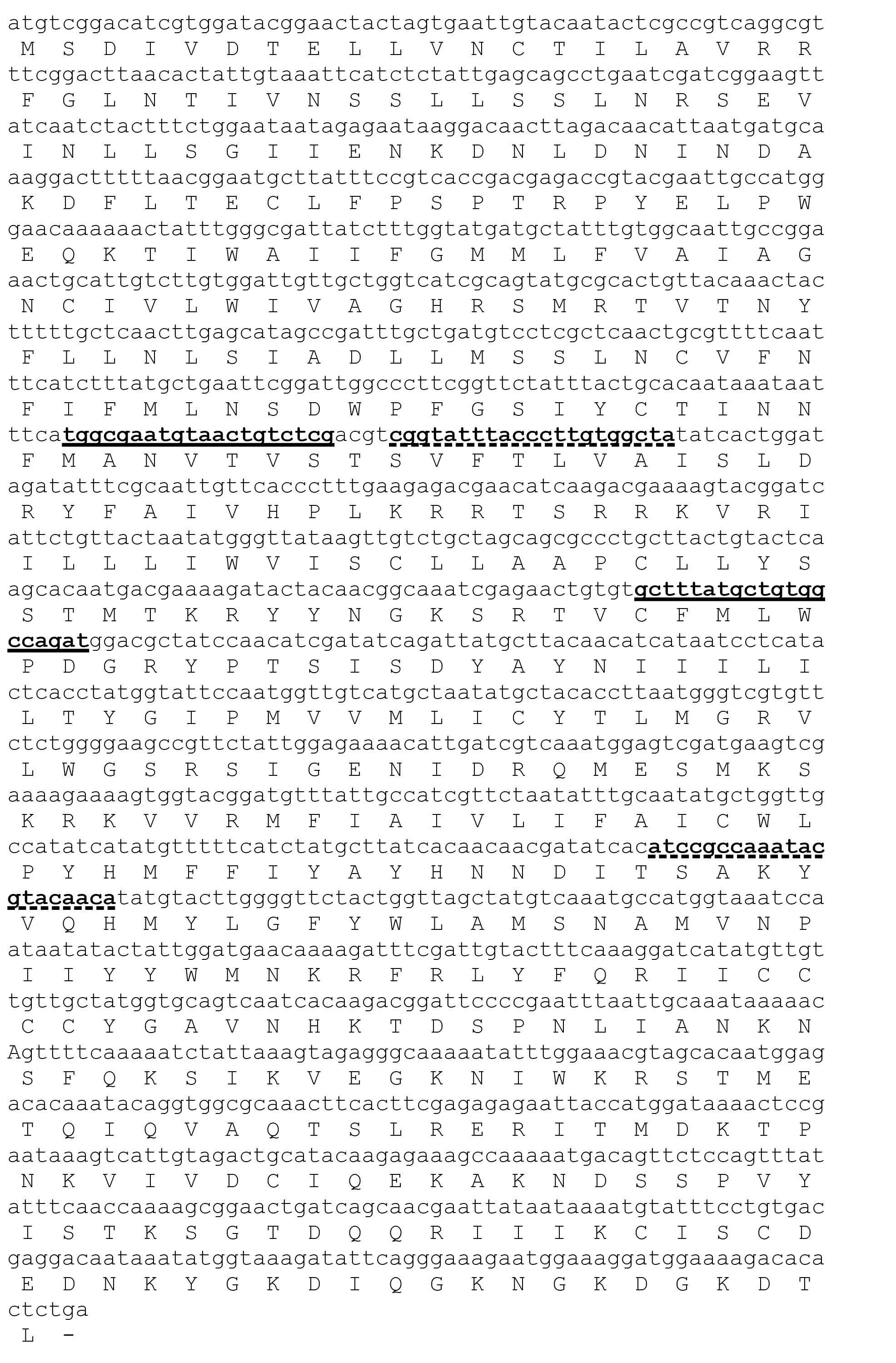

Supplement: S1 Fig — The annealing sites of the qRT-PCR primers are denoted in bold and underlined, while the annealing sites of the primers for the RNAi-constructs are denoted in bold and dashed underlined in the cDNA sequence. (JPG) [file pone.0193058.s001.jpg]
